# Supplementary material for: Evaluating the effectiveness of concurrent opioid agonist treatment and physician-based mental health services for patients with mental disorders in Ontario, Canada
Source: PLoS One. 2020 Dec 18;15(12):e0243317. doi: 10.1371/journal.pone.0243317 (PMC7748134; doi:10.1371/journal.pone.0243317)
Supplement: S1 Table — (DOCX) [file pone.0243317.s001.docx]

S1 Table- Definition and ICD 9 and ICD10 codes for Mental Health Conditions

| Definition Mental Health Conditions | | |
| --- | --- | --- |
| **Mental Health Conditions** | **ICD-10 Codes (DX10CODE1)** | **ICD-9 Codes** [Go Back](#APPENDIX_1_back) |
| **Neurodevelopmental Disorders** | **F90** **Attention-deficit hyperactivity disorders**  **Includes:**  attention deficit disorder with hyperactivity  attention deficit syndrome with hyperactivity  **F90.0 Attention-deficit hyperactivity disorder, predominantly inattentive type**  **F90.1 Attention-deficit hyperactivity disorder, predominantly hyperactive type**  **F90.2 Attention-deficit hyperactivity disorder, combined type**  **F90.8 Attention-deficit hyperactivity disorder, other type**  **F90.9 Attention-deficit hyperactivity disorder, unspecified type**  Attention-deficit hyperactivity disorder of childhood or adolescence NOS  Attention-deficit hyperactivity disorder NOS | **314.0 ATTENTION DEFICIT DIS***  314.00 Attention-Deficit/Hyperactivity Disorder, Predominantly Inattentive Type  314.01 Attention-Deficit/Hyperactivity Disorder, Combined Type or Attention-Deficit/Hyperactivity Disorder Predominantly Hyperactive-Impulsive Type  314.9 Attention-Deficit/Hyperactivity Disorder NOS |
| **Schizophrenia Spectrum**  **and Related Disorders** | **Schizophrenia, schizotypal, delusional, and other non-mood psychotic disorders (F20-F29)**  **F20 Schizophrenia**  **F20.0 Paranoid schizophrenia** Paraphrenic schizophrenia  **F20.1 Disorganized schizophrenia**  Hebephrenic schizophrenia  Hebephrenia  **F20.2 Catatonic schizophrenia**  Schizophrenic catalepsy  Schizophrenic catatonia  Schizophrenic flexibilitas cerea  **F20.3** **Undifferentiated schizophrenia**  Atypical schizophrenia  **F20.5 Residual schizophrenia**  Restzustand (schizophrenic)  Schizophrenic residual state  **F20.8 Other schizophrenia**  **F20.81 Schizophreniform disorder**  Schizophreniform psychosis NOS  **F20.89 Other schizophrenia**  Cenesthopathic schizophrenia  Simple schizophrenia  **F20.9 Schizophrenia, unspecified**  **F21 Schizotypal disorder**  Borderline schizophrenia  Latent schizophrenia  Latent schizophrenic reaction  Prepsychotic schizophrenia  Prodromal schizophrenia  Pseudoneurotic schizophrenia  Pseudopsychopathic schizophrenia  Schizotypal personality disorder  **F22 Delusional disorders**  Delusional dysmorphophobia  Involutional paranoid state  Paranoia  Paranoia querulans  Paranoid psychosis  Paranoid state  Paraphrenia (late)  Sensitiver Beziehungswahn  **F23 Brief psychotic disorder**  Paranoid reaction  Psychogenic paranoid psychosis  **F24 Shared psychotic disorder**  Folie à deux  Induced paranoid disorder  Induced psychotic disorder  **F25 Schizoaffective disorders**  **F25.0 Schizoaffective disorder, bipolar type**  Cyclic schizophrenia  Schizoaffective disorder, manic type  Schizoaffective disorder, mixed type  Schizoaffective psychosis, bipolar type  Schizophreniform psychosis, manic type  **F25.1 Schizoaffective disorder, depressive type**  Schizoaffective psychosis, depressive type  Schizophreniform psychosis, depressive type  **F25.8 Other schizoaffective disorders**  **F25.9 Schizoaffective disorder, unspecified**  Schizoaffective psychosis NOS  **F28 Other psychotic disorder not due to a substance or known physiological condition**  Chronic hallucinatory psychosis  **F29 Unspecified psychosis not due to a substance or known physiological condition**  Psychosis NOS | **295 SCHIZOPHRENIC DISORDERS***  295.0 SIMPLE SCHIZOPHRENIA*  295.00 SIMPLE SCHIZOPHREN-UNSPECIFIED  295.01 SIMPL SCHIZOPHREN-SUBCHR  295.02 SIMPLE SCHIZOPHREN-CHR  295.03 SIMP SCHIZ-SUBCHR/EXACER  295.04 SIMPL SCHIZO-CHR/EXACERB  295.05 SIMPL SCHIZOPHREN-REMISS  295.1 HEBEPHRENIA*  295.10 Schizophrenia, Disorganized Type  295.11 HEBEPHRENIA-SUBCHRONIC  295.12 HEBEPHRENIA-CHRONIC  295.13 HEBEPHREN-SUBCHR/EXACERB  295.14 HEBEPHRENIA-CHR/EXACERB  295.15 HEBEPHRENIA-REMISSION  295.2 CATATONIC SCHIZOPHRENIA*  295.20 Schizophrenia, Catatonic Type  295.21 CATATONIA-SUBCHRONIC  295.22 CATATONIA-CHRONIC  295.23 CATATONIA-SUBCHR/EXACERB  295.24 CATATONIA-CHR/EXACERB  295.25 CATATONIA-REMISSION  295.3 PARANOID SCHIZOPHRENIA*  295.30 Schizophrenia, Paranoid Type  295.31 PARANOID SCHIZO-SUBCHR  295.32 PARANOID SCHIZO-CHRONIC  295.33 PARAN SCHIZO-SUBCHR/EXAC  295.34 PARAN SCHIZO-CHR/EXACERB  295.35 PARANOID SCHIZO-REMISS  295.4 AC SCHIZOPHRENIC EPISODE*  295.40 Schizophreniform Disorder  295.41 AC SCHIZOPHRENIA-SUBCHR  295.42 AC SCHIZOPHRENIA-CHR  295.43 AC SCHIZO-SUBCHR/EXACERB  295.44 AC SCHIZOPHR-CHR/EXACERB  295.45 AC SCHIZOPHRENIA-REMISS  295.5 LATENT SCHIZOPHRENIA*  295.50 LATENT SCHIZOPHREN-UNSP  295.51 LAT SCHIZOPHREN-SUBCHR  295.52 LATENT SCHIZOPHREN-CHR  295.53 LAT SCHIZO-SUBCHR/EXACER  295.54 LATENT SCHIZO-CHR/EXACER  295.55 LAT SCHIZOPHREN-REMISS  295.6 RESIDUAL SCHIZOPHRENIA*  295.60 Schizophrenia, Residual Type  295.61 RESID SCHIZOPHREN-SUBCHR  295.62 RESIDUAL SCHIZOPHREN-CHR  295.63 RESID SCHIZO-SUBCHR/EXAC  295.64 RESID SCHIZO-CHR/EXACERB  295.65 RESID SCHIZOPHREN-REMISS  295.7 SCHIZOAFFECTIVE TYPE*  295.70 Schizoaffective Disorder  295.71 SCHIZOAFFECTIVE-SUBCHR  295.72 SCHIZOAFFECTIVE-CHRONIC  295.73 SCHIZOAFF-SUBCHR/EXACER  295.74 SCHIZOAFFECT-CHR/EXACER  295.75 SCHIZOAFFECTIVE-REMISS  295.8 SCHIZOPHRENIA NEC*  295.80 SCHIZOPHRENIA NEC-UNSPEC  295.81 SCHIZOPHRENIA NEC-SUBCHR  295.82 SCHIZOPHRENIA NEC-CHR  295.83 SCHIZO NEC-SUBCHR/EXACER  295.84 SCHIZO NEC-CHR/EXACERB  295.85 SCHIZOPHRENIA NEC-REMISS  295.9 SCHIZOPHRENIA NOS*  295.90 Schizophrenia Undifferentiated Type  295.91 SCHIZOPHRENIA NOS-SUBCHR  295.92 SCHIZOPHRENIA NOS-CHR  295.93 SCHIZO NOS-SUBCHR/EXACER  295.94 SCHIZO NOS-CHR/EXACERB  295.95 SCHIZOPHRENIA NOS-REMISS  297.1 Delusional Disorder  297.3 Shared Psychotic Disorder  298.0 REACT DEPRESS PSYCHOSIS  298.1 EXCITATIV TYPE PSYCHOSIS  298.2 REACTIVE CONFUSION  298.3 ACUTE PARANOID REACTION  298.4 PSYCHOGEN PARANOID PSYCH  298.8 Brief Psychotic Disorder  298.9 Psychotic Disorder NOS |
| **Bipolar and Related Disorders** | **F30 –F31 Manic episode**  **Includes:**  bipolar disorder, single manic episode  mixed affective episode  **F30.1 Manic episode without psychotic symptoms**  **F30.10 Manic episode without psychotic symptoms, unspecified**  **F30.11 Manic episode without psychotic symptoms, mild**  **F30.12 Manic episode without psychotic symptoms, moderate**  **F30.13 Manic episode, severe, without psychotic symptoms**  **F30.2 Manic episode, severe with psychotic symptoms**  Manic stupor  Mania with mood-congruent psychotic symptoms  Mania with mood-incongruent psychotic symptoms  **F30.3 Manic episode in partial remission**  **F30.4 Manic episode in full remission**  **F30.8 Other manic episodes**  Hypomania  **F30.9 Manic episode, unspecified**  Mania NOS  **F31 Bipolar disorder**  **Includes:**  manic-depressive illness  manic-depressive psychosis  manic-depressive reaction  **F31.0 Bipolar disorder, current episode hypomanic**  **F31.1 Bipolar disorder, current episode manic without psychotic features**  **F31.10 Bipolar disorder, current episode manic without psychotic features, unspecified**  **F31.11 Bipolar disorder, current episode manic without psychotic features, mild**  **F31.12 Bipolar disorder, current episode manic without psychotic features, moderate**  **F31.13 Bipolar disorder, current episode manic without psychotic features, severe**  **F31.2 Bipolar disorder, current episode manic severe with psychotic features** Bipolar disorder, current episode manic with mood-congruent psychotic symptoms  Bipolar disorder, current episode manic with mood-incongruent psychotic symptoms  F31.3 **Bipolar disorder, current episode depressed, mild or moderate severity**  **F31.30 Bipolar disorder, current episode depressed, mild or moderate severity, unspecified**  **F31.31 Bipolar disorder, current episode depressed, mild**  **F31.32 Bipolar disorder, current episode depressed, moderate**  **F31.4 Bipolar disorder, current episode depressed, severe, without psychotic features**  **F31.5 Bipolar disorder, current episode depressed, severe, with psychotic features**  Bipolar disorder, current episode depressed with mood-incongruent psychotic symptoms  Bipolar disorder, current episode depressed with mood-congruent psychotic symptoms  **F31.6 Bipolar disorder, current episode mixed**  **F31.60 Bipolar disorder, current episode mixed, unspecified**  **F31.61 Bipolar disorder, current episode mixed, mild**  **F31.62 Bipolar disorder, current episode mixed, moderate**  **F31.63 Bipolar disorder, current episode mixed, severe, without psychotic features**  **F31.64 Bipolar disorder, current episode mixed, severe, with psychotic features**  Bipolar disorder, current episode mixed with mood-congruent psychotic symptoms  Bipolar disorder, current episode mixed with mood-incongruent psychotic symptoms  **F31.7 Bipolar disorder, currently in remission**  **F31.70 Bipolar disorder, currently in remission, most recent episode unspecified**  **F31.71 Bipolar disorder, in partial remission, most recent episode hypomanic**  **F31.72 Bipolar disorder, in full remission, most recent episode hypomanic**  **F31.73 Bipolar disorder, in partial remission, most recent episode manic**  **F31.74 Bipolar disorder, in full remission, most recent episode manic**  **F31.75 Bipolar disorder, in partial remission, most recent episode depressed**  **F31.76 Bipolar disorder, in full remission, most recent episode depressed**  **F31.77 Bipolar disorder, in partial remission, most recent episode mixed**  **F31.78 Bipolar disorder, in full remission, most recent episode mixed**  **F31.8 Other bipolar disorders**  **F31.81 Bipolar II disorder**  **F31.89 Other bipolar disorder**  Recurrent manic episodes NOS  **F31.9 Bipolar disorder, unspecified** | 296.00 Bipolar I Disorder, Single Manic Episode, Unspecified  296.01 Bipolar I Disorder, Single Manic Episode, Mild  296.02 Bipolar I Disorder, Single Manic Episode, Moderate  296.03 Bipolar I Disorder, Single Manic Episode, Severe Without Psychotic Features  296.04 Bipolar I Disorder, Single Manic Episode, Severe With Psychotic Features  296.05 Bipolar I Disorder, Single Manic Episode, In Partial Remission  296.06 Bipolar I Disorder, Single Manic Episode, In Full Remission  296.1 MANIC, RECURRENT EPISODE*  296.10 RECUR MANIC DIS-UNSPEC  296.11 RECUR MANIC DIS-MILD  296.12 RECUR MANIC DIS-MOD  296.13 RECUR MANIC DIS-SEVERE  296.14 RECUR MANIC-SEV W PSYCHO  296.15 RECUR MANIC-PART REMISS  296.16 RECUR MANIC-FULL REMISS  296.4 BIPOLAR AFFECTIVE, MANIC*  296.40 Bipolar I Disorder, Most Recent Episode Hypomanic or Manic, Unspecified  296.41 Bipolar I Disorder, Most Recent Episode Manic, Mild  296.42 Bipolar I Disorder, Most Recent Episode Manic, Moderate  296.43 Bipolar I Disorder, Most Recent Episode Manic, Severe Without Psychotic Features  296.44 Bipolar I Disorder, Most Recent Episode Manic, Severe With Psychotic Features  296.45 Bipolar I Disorder, Most Recent Episode Manic, In Partial Remission  296.46 Bipolar I Disorder, Most Recent Episode Manic, In Full Remission  296.5 BIPOLAR AFFECT, DEPRESS*  296.50 Bipolar I Disorder, Most Recent Episode Depressed, Unspecified  296.51 Bipolar I Disorder, Most Recent Episode Depressed, Mild  296.52 Bipolar I Disorder, Most Recent Episode Depressed, Moderate  296.53 Bipolar I Disorder, Most Recent Episode Depressed, Severe Without Psychotic Features  296.54 Bipolar I Disorder, Most Recent Episode Depressed, Severe With Psychotic Features  296.55 Bipolar I Disorder, Most Recent Episode Depressed, In Partial Remission  296.56 Bipolar I Disorder, Most Recent Episode Depressed, In Full Remission  296.6 BIPOLAR AFFECTIVE, MIXED*  296.60 Bipolar I Disorder, Most Recent Episode Mixed, Unspecified  296.61 Bipolar I Disorder, Most Recent Episode Mixed, Mild  296.62 Bipolar I Disorder, Most Recent Episode Mixed, Moderate  296.63 Bipolar I Disorder, Most Recent Episode Mixed, Severe Without Psychotic Features  296.64 Bipolar I Disorder, Most Recent Episode Mixed, Severe With Psychotic Features  296.65 Bipolar I Disorder, Most Recent Episode Mixed, In Partial Remission  296.66 Bipolar I Disorder, Most Recent Episode Mixed, In Full Remission  296.7 Bipolar I Disorder, Most Recent Episode Unspecified  296.8 MANIC-DEPRESSIVE NEC/NOS*  296.80 Bipolar Disorder NOS  296.81 ATYPICAL MANIC DISORDER  296.82 ATYPICAL DEPRESSIVE DIS  296.89 Bipolar II Disorder |
| **Depressive Disorders** | **F32 Major depressive disorder, single episode**  **Includes:**  single episode of agitated depression  single episode of depressive reaction  single episode of major depression  single episode of psychogenic depression  single episode of reactive depression  single episode of vital depression  **F32.0 Major depressive disorder, single episode, mild**  **F32.1 Major depressive disorder, single episode, moderate**  **F32.2 Major depressive disorder, single episode, severe without psychotic features**  **F32.3Major depressive disorder, single episode, severe with psychotic features**  Single episode of major depression with mood-congruent psychotic symptoms  Single episode of major depression with mood-incongruent psychotic symptoms  Single episode of major depression with psychotic symptoms  Single episode of psychogenic depressive psychosis  Single episode of psychotic depression  Single episode of reactive depressive psychosis  **F32.4 Major depressive disorder, single episode, in partial remission**  **F32.5 Major depressive disorder, single episode, in full remission**  **F32.8 Other depressive episodes**  Atypical depression  Post-schizophrenic depression  Single episode of 'masked' depression NOS  **F32.9 Major depressive disorder, single episode, unspecified**  Depression NOS  Depressive disorder NOS  Major depression NOS  **F33 Major depressive disorder, recurrent**  **Includes:**  recurrent episodes of depressive reaction  recurrent episodes of endogenous depression  recurrent episodes of major depression  recurrent episodes of psychogenic depression  recurrent episodes of reactive depression  recurrent episodes of seasonal depressive disorder  recurrent episodes of vital depression  **F33.0 Major depressive disorder, recurrent, mild**  **F33.1 Major depressive disorder, recurrent, moderate**  **F33.2 Major depressive disorder, recurrent severe without psychotic features**  **F33.3 Major depressive disorder, recurrent, severe with psychotic symptoms**  Endogenous depression with psychotic symptoms  Recurrent severe episodes of major depression with mood-congruent psychotic symptoms  Recurrent severe episodes of major depression with mood-incongruent psychotic symptoms  Recurrent severe episodes of major depression with psychotic symptoms  Recurrent severe episodes of psychogenic depressive psychosis  Recurrent severe episodes of psychotic depression  Recurrent severe episodes of reactive depressive psychosis  **F33.4 Major depressive disorder, recurrent, in remission**  **F33.40 Major depressive disorder, recurrent, in remission, unspecified**  **F33.41 Major depressive disorder, recurrent, in partial remission**  **F33.42 Major depressive disorder, recurrent, in full remission**  **F33.8 Other recurrent depressive disorders**  **F33.9 Major depressive disorder, recurrent, unspecified**  Monopolar depression NOS  **F34 Persistent mood [affective] disorders**  **F34.0 Cyclothymic disorder**  Affective personality disorder  Cycloid personality  Cyclothymia  Cyclothymic personality  **F34.1 Dysthymic disorder**  Depressive neurosis  Depressive personality disorder  Dysthymia  Neurotic depression  Persistent anxiety depression  **F34.8 Other persistent mood [affective] disorders**  **F34.9 Persistent mood [affective] disorder, unspecified**  **F39 Unspecified mood [affective] disorder**  Affective psychosis NOS | 296.2 DEPR PSYCH, SINGL EPISOD*  296.20 Major Depressive Disorder, Single Episode, Unspecified  296.21 Major Depressive Disorder, Single Episode, Mild  296.22 Major Depressive Disorder, Single Episode, Moderate  296.23 Major Depressive Disorder, Single Episode, Severe Without Psychotic Features  296.24 Major Depressive Disorder, Single Episode, Severe With Psychotic Features  296.25 Major Depressive Disorder, Single Episode, In Partial Remission  296.26 Major Depressive Disorder, Single Episode, In Full Remission  296.3 DEPR PSYCH, RECUR EPISOD*  296.30 Major Depressive Disorder, Recurrent, Unspecified  296.31 Major Depressive Disorder, Recurrent, Mild  296.32 Major Depressive Disorder, Recurrent, Moderate  296.33 Major Depressive Disorder, Recurrent, Severe Without Psychotic Features  296.34 Major Depressive Disorder, Recurrent, Severe With Psychotic Features  296.35 Major Depressive Disorder, Recurrent, In Partial Remission  296.36 Major Depressive Disorder, Recurrent, In Full Remission  296.90 Mood Disorder NOS  300.4 Dysthymic Disorder  311 Depressive Disorder NOS |
| **Anxiety Disorders** | **F40 Phobic anxiety disorders**  **F40.0 Agoraphobia**  **F40.00 Agoraphobia, unspecified**  **F40.01 Agoraphobia with panic disorder**  Panic disorder with agoraphobia  **F40.02 Agoraphobia without panic disorder**  **F40.1 Social phobias**  Anthropophobia  Social anxiety disorder of childhood  **F40.10 Social phobia, unspecified**  **F40.11 Social phobia, generalized**  **F41.0 Panic disorder [episodic paroxysmal anxiety] without agoraphobia**  Panic attack  Panic state  **F41.1Generalized anxiety disorder**  Anxiety neurosis  Anxiety reaction  Anxiety state  Overanxious disorder  **F41.3Other mixed anxiety disorders**  **F41.8 Other specified anxiety disorders**  Anxiety depression (mild or not persistent)  Anxiety hysteria  Mixed anxiety and depressive disorder  **F41.9 Anxiety disorder, unspecified**  Anxiety NOS | 300.0 ANXIETY STATES*  300.00 Anxiety Disorder NOS  300.01 Panic Disorder Without Agoraphobia  300.02 Generalized Anxiety Disorder  300.09 ANXIETY STATE NEC  300.2 PHOBIC DISORDERS*  300.20 PHOBIA NOS  300.21 Panic Disorder With Agoraphobia  300.22 Agoraphobia Without History of Panic Disorder  300.23 Social Phobia |
| **Obsessive-Compulsive**  **and Related Disorders** | **F42 Obsessive-compulsive disorder**  Anancastic neurosis  Obsessive-compulsive neurosis | 300.3 Obsessive-Compulsive Disorder  312.39 Trichotillomania |
| **Trauma and Stressor-Related**  **Disorders** | F43 **Reaction to severe stress, and adjustment disorders**  **F43.0 Acute stress reaction**  Acute crisis reaction  Acute reaction to stress  Combat and operational stress reaction  Combat fatigue  Crisis state  Psychic shock  **F43.1 Post-traumatic stress disorder (PTSD)**  Traumatic neurosis  **F43.10 Post-traumatic stress disorder, unspecified**  **F43. 11 Post-traumatic stress disorder, acute**  **F43.12 Post-traumatic stress disorder, chronic**  **F43.2 Adjustment disorders**  Culture shock  Grief reaction  Hospitalism in children  **F43.20 Adjustment disorder, unspecified**  **F43.21 Adjustment disorder with depressed mood**  **F43.22 Adjustment disorder with anxiety**  **F43.23 Adjustment disorder with mixed anxiety and depressed mood**  **F43.24 Adjustment disorder with disturbance of conduct**  **F43.25 Adjustment disorder with mixed disturbance of emotions and conduct**  **F43.29 Adjustment disorder with other symptoms**  **F43.8 Other reactions to severe stress**  **F43.9 Reaction to severe stress, unspecified** | **308 Acute reaction to stress**  **309 Adjustment reaction**  308 ACUTE REACTION TO STRESS*  308.0 STRESS REACT, EMOTIONAL  308.1 STRESS REACTION, FUGUE  308.2 STRESS REACT, PSYCHOMOT  308.3 Acute Stress Disorder  308.4 STRESS REACT, MIXED DIS  308.9 ACUTE STRESS REACT NOS  309 ADJUSTMENT REACTION*  309.0 Adjustment Disorder With Depressed Mood  309.1 PROLONG DEPRESSIVE REACT  309.2 ADJUST REACT/OTH EMOTION*  309.21 Separation Anxiety Disorder  309.22 EMANCIPATION DISORDER  309.23 ACADEMIC/WORK INHIBITION  309.24 Adjustment Disorder With Anxiety  309.28 Adjustment Disorder With Mixed Anxiety and Depressed Mood  309.29 ADJ REACT-EMOTION NEC  309.3 Adjustment Disorder With Disturbance of Conduct  309.4 Adjustment Disorder With Mixed Disturbance of Emotions and Conduct  309.8 OTHER ADJUST REACTION*  **309.81 Posttraumatic Stress Disorder**  309.82 ADJUST REACT-PHYS SYMPT  309.83 ADJUST REACT-WITHDRAWAL  309.89 ADJUSTMENT REACTION NEC  309.9 Adjustment Disorder Unspecified |
| **Feeding and Eating Disorders** | **F50 Eating disorders**  **F50.00Anorexia nervosa**  F50.01**Anorexia nervosa, unspecified**  **F50.02 Anorexia nervosa, restricting type**  **F50.03 Anorexia nervosa, binge eating/purging type**  **F50.2 Bulimia nervosa**  Bulimia NOS  Hyperorexia nervosa  **F50.9 Eating disorder, unspecified**  Atypical anorexia nervosa  Atypical bulimia nervosa | 307.1 Anorexia Nervosa  307.5 EATING DISORDERS NEC/NOS*  307.50 Eating Disorder NOS  307.51 Bulimia Nervosa |
| **Gender Dysphoria** | **F64 Gender identity disorders**  **F64.1 Gender identity disorder in adolescence and adulthood**  Dual role transvestism  Transsexualism  **F64.2 Gender identity disorder of childhood**  **F64.8 Other gender identity disorders**  **F64.9 Gender identity disorder, unspecified**  Gender-role disorder NOS | 302.5 TRANS-SEXUALISM*  302.50 TRANS-SEXUALISM NOS  302.51 TRANS-SEXUALISM, ASEXUAL  302.52 TRANS-SEXUAL, HOMOSEXUAL  302.53 TRANS-SEX, HETEROSEXUAL  302.6 Gender Identity Disorder in Children or Gender Identity Disorder NOS  302.85 Gender Identity Disorder in Adolescents or Adults |
| **Disruptive, Impulse-Control,**  **and Conduct Disorders** | **Behavioral and emotional disorders with onset usually occurring in childhood and adolescence (F90-F98)**  Codes within categories F90-F98 may be used regardless of the age of a patient. These disorders generally have onset within the childhood or adolescent years, but may continue throughout life or not be diagnosed until adulthood  **F91 Conduct disorders**  **F91.0 Conduct disorder confined to family context**  **F91.1 Conduct disorder, childhood-onset type**  Unsocialized conduct disorder  Conduct disorder, solitary aggressive type  Unsocialized aggressive disorder  **F91.2 Conduct disorder, adolescent-onset type**  Socialized conduct disorder  Conduct disorder, group type  **F91.3 Oppositional defiant disorder**  **F91.8 Other conduct disorders**  **F91.9 Conduct disorder, unspecified**  Behavioral disorder NOS  Conduct disorder NOS  Disruptive behavior disorder NOS  **F63 Impulse disorders**  **Excludes2:**  habitual excessive use of alcohol or psychoactive substances (F10-F19)  impulse disorders involving sexual behavior (F65.-)  **F63.0 Pathological gambling**  Compulsive gambling  **F63.1 Pyromania**  Pathological fire-setting  **F63.2 Kleptomania**  Pathological stealing  **F63.3 Trichotillomania**  Hair plucking  **F63.8 Other impulse disorders**  **F63.81 Intermittent explosive disorder**  **F63.89 Other impulse disorders**  **F63.9 Impulse disorder, unspecified**  Impulse control disorder NOS | 312 Disturbance of conduct, not elsewhere classified  312.3 IMPULSE CONTROL DIS NEC*  312.30 Impulse-Control Disorder NOS  312.81 Conduct Disorder, Childhood-Onset Type  312.82 Conduct Disorder, Adolescent-Onset Type  312.89 Conduct Disorder, Unspecified Onset  312.9 Disruptive Behavior Disorder NOS  312.20 SOCIAL CONDUCT DIS-UNSP  312.21 SOCIAL CONDUCT DIS-MILD  312.22 SOCIAL CONDUCT DIS-MOD  312.23 SOCIAL CONDUCT DIS-SEV  312.3 IMPULSE CONTROL DIS NEC*  312.30 Impulse-Control Disorder NOS  312.31 Pathological Gambling  312.32 Kleptomania  312.33 Pyromania  312.34 Intermittent Explosive Disorder  312.35 ISOLATED EXPLOSIVE DIS  313.81 Oppositional Defiant Disorder |
| **Personality Disorders** | **F60**  **F60.1 Schizoid personality disorder**  **F60.2 Antisocial personality disorder**  Amoral personality (disorder)  Asocial personality (disorder)  Dissocial personality disorder  Psychopathic personality (disorder)  Sociopathic personality (disorder)  **F60.3 Borderline personality disorder**  Aggressive personality (disorder)  Emotionally unstable personality disorder  Explosive personality (disorder)  **F60.4 Histrionic personality disorder**  Hysterical personality (disorder)  Psychoinfantile personality (disorder)  **F60.5 Obsessive-compulsive personality disorder**  Anankastic personality (disorder)  Compulsive personality (disorder)  Obsessional personality (disorder)  obsessive-compulsive disorder (F42)  **F60.6 Avoidant personality disorder**  Anxious personality disorder  **F60.7 Dependent personality disorder**  Asthenic personality (disorder)  Inadequate personality (disorder)  Passive personality (disorder)  **F60.8 Other specific personality disorders**  **F60.81 Narcissistic personality disorder**  **F60.89 Other specific personality disorders**  Eccentric personality disorder  'Haltlose' type personality disorder  Immature personality disorder  Passive-aggressive personality disorder  Psychoneurotic personality disorder  Self-defeating personality disorder  **F60.9 Personality disorder, unspecified**  Character disorder NOS  Character neurosis NOS  Pathological personality NOS | **301 PERSONALITY DISORDERS***  301.0 Paranoid Personality Disorder  301.1 AFFECTIVE PERSONALITY*  301.10 AFFECTIV PERSONALITY NOS  301.11 CHRONIC HYPOMANIC PERSON  301.12 CHR DEPRESSIVE PERSON  301.13 Cyclothymic Disorder  301.2 SCHIZOID PERSONALITY*  301.20 Schizoid Personality Disorder  301.21 INTROVERTED PERSONALITY  301.22 Schizotypal Personality Disorder  301.3 EXPLOSIVE PERSONALITY  301.4 Obsessive-Compulsive Personality Disorder  301.50 Histrionic Personality Disorder  301.51 CHR FACTITIOUS ILLNESS  301.59 HISTRIONIC PERSON NEC  301.6 Dependent Personality Disorder  301.7 Antisocial Personality Disorder  301.8 OTHER PERSONALITY DIS*  301.81 Narcissistic Personality Disorder  301.82 Avoidant Personality Disorder  301.83 Borderline Personality Disorder  301.84 PASSIVE-AGGRESSIV PERSON  301.89 PERSONALITY DISORDER NEC  301.9 Personality Disorder NOS |
| **Substance Use Disorders** | \| **F10-F19** \|  \|  \|  \|  \|  \| \| --- \| --- \| --- \| --- \| --- \| --- \| \|  \|  \|  \|  \| \|  \|  \|  \|  \| \|  \|  \|  \|  \|  \|  \| \|  \|  \|  \|  \|  \| \|  \|  \|  \|  \|  \| \|  \|  \|  \|  \| \|  \|  \|  \| \|  \|  \|  \|  \|  \|  \| \|  \|  \|  \|  \| \|  \|  \|  \|  \|  \|  \| \|  \|  \|  \| \|  \|  \|  \|  \|  \|  \| \|  \|  \| \|  \|  \|  \|  \|  \|  \| \|  \|  \|  \| \|  \|  \|  \|  \|  \|  \| \|  \|  \| \|  \|  \|  \|  \|  \|  \| \|  \|  \| \|  \|  \|  \|  \|  \|  \| \|  \| \|  \|  \|  \|  \|  \|  \| \|  \|  \|  \|  \|  \|  \| \|  \|  \|  \|  \|  \|  \| \|  \|  \|  \|  \|  \|  \|  \| | **303 Alcohol dependence syndrome**  **304 Drug dependence**  **305 Nondependent abuse of drugs**  303 ALCOHOL DEPENDENCE SYNDR*  303.0 AC ALCOHOL INTOXICATION*  303.00 Alcohol Intoxication  303.01 AC ALCOHOL INTOX-CONTIN  303.02 AC ALCOHOL INTOX-EPISOD  303.03 AC ALCOHOL INTOX-REMISS  303.9 ALCOHOL DEPEND NEC/NOS*  303.90 Alcohol Dependence  303.91 ALCOH DEP NEC/NOS-CONTIN  303.92 ALCOH DEP NEC/NOS-EPISOD  303.93 ALCOH DEP NEC/NOS-REMISS  304 DRUG DEPENDENCE*  304.0 OPIOID TYPE DEPENDENCE*  304.00 Opioid Dependence  304.01 OPIOID DEPEND-CONTIN  304.02 OPIOID DEPENDENCE-EPISOD  304.03 OPIOID DEPENDENCE-REMISS  304.1 BARBITURATE DEPENDENCE*  304.10 Sedative, Hypnotic, or Anxiolytic Dependence  304.11 BARBITURAT DEPEND-CONTIN  304.12 BARBITURAT DEPEND-EPISOD  304.13 BARBITURAT DEPEND-REMISS  304.2 COCAINE DEPENDENCE*  304.20 Cocaine Dependence  304.21 COCAINE DEPEND-CONTIN  304.22 COCAINE DEPEND-EPISODIC  304.23 COCAINE DEPEND-REMISS  304.3 CANNABIS DEPENDENCE*  304.30 Cannabis Dependence  304.31 CANNABIS DEPEND-CONTIN  304.32 CANNABIS DEPEND-EPISODIC  304.33 CANNABIS DEPEND-REMISS  304.4 AMPHETAMINE DEPENDENCE*  304.40 Amphetamine Dependence  304.41 AMPHETAMIN DEPEND-CONTIN  304.42 AMPHETAMIN DEPEND-EPISOD  304.43 AMPHETAMIN DEPEND-REMISS  304.5 HALLUCINOGEN DEPENDENCE*  304.50 Hallucinogen Dependence  304.51 HALLUCINOGEN DEP-CONTIN  304.52 HALLUCINOGEN DEP-EPISOD  304.53 HALLUCINOGEN DEP-REMISS  304.6 DRUG DEPENDENCE NEC*  304.60 Inhalant Dependence or Phencyclidine Dependence  304.61 DRUG DEPEND NEC-CONTIN  304.62 DRUG DEPEND NEC-EPISODIC  304.63 DRUG DEPEND NEC-IN REM  304.7 OPIOID/OTHER DRUG DEPEND*  304.70 OPIOID/OTHER DEP-UNSPEC  304.71 OPIOID/OTHER DEP-CONTIN  304.72 OPIOID/OTHER DEP-EPISOD  304.73 OPIOID/OTHER DEP-REMISS  304.8 COMB DRUG DEPENDENCE NEC*  304.80 Polysubstance Dependence  304.81 COMB DRUG DEP NEC-CONTIN  304.82 COMB DRUG DEP NEC-EPISOD  304.83 COMB DRUG DEP NEC-REMISS  304.9 DRUG DEPENDENCE NOS*  304.90 Other (or Unknown) Substance Dependence  304.91 DRUG DEPEND NOS-CONTIN  304.92 DRUG DEPEND NOS-EPISODIC  304.93 DRUG DEPEND NOS-REMISS  305 NONDEPENDENT DRUG ABUSE*  305.0 ALCOHOL ABUSE*  305.00 Alcohol Abuse  305.01 ALCOHOL ABUSE-CONTINUOUS  305.02 ALCOHOL ABUSE-EPISODIC  305.03 ALCOHOL ABUSE-IN REMISS  305.1 Nicotine Dependence  305.2 CANNABIS ABUSE*  305.20 Cannabis Abuse  305.21 CANNABIS ABUSE-CONTIN  305.22 CANNABIS ABUSE-EPISODIC  305.23 CANNABIS ABUSE-IN REMISS  305.3 HALLUCINOGEN ABUSE*  305.30 Hallucinogen Abuse  305.31 HALLUCINOG ABUSE-CONTIN  305.32 HALLUCINOG ABUSE-EPISOD  305.33 HALLUCINOG ABUSE-REMISS  305.4 BARBITURATE ABUSE*  305.40 Sedative, Hypnotic, or Anxiolytic Abuse  305.41 BARBITURATE ABUSE-CONTIN  305.42 BARBITURATE ABUSE-EPISOD  305.43 BARBITURATE ABUSE-REMISS  305.5 OPIOID ABUSE*  305.50 Opioid Abuse  305.51 OPIOID ABUSE-CONTINUOUS  305.52 OPIOID ABUSE-EPISODIC  305.53 OPIOID ABUSE-IN REMISS  305.6 COCAINE ABUSE*  305.60 Cocaine Abuse  305.61 COCAINE ABUSE-CONTINUOUS  305.62 COCAINE ABUSE-EPISODIC  305.63 COCAINE ABUSE-IN REMISS  305.7 AMPHETAMINE ABUSE*  305.70 Amphetamine Abuse  305.71 AMPHETAMINE ABUSE-CONTIN  305.72 AMPHETAMINE ABUSE-EPISOD  305.73 AMPHETAMINE ABUSE-REMISS  305.8 ANTIDEPRESSANT ABUSE*  305.80 ANTIDEPRESS ABUSE-UNSPEC  305.81 ANTIDEPRESS ABUSE-CONTIN  305.82 ANTIDEPRESS ABUSE-EPISOD  305.83 ANTIDEPRESS ABUSE-REMISS  305.9 DRUG ABUSE NEC/NOS*  305.90 Phencyclidine Abuse, Inhalant Abuse, Other (or Unknown) Substance Abuse  305.91 DRUG ABUSE NEC-CONTIN  305.92 DRUG ABUSE NEC-EPISODIC  305.93 DRUG ABUSE NEC-IN REMISS  291.3 Alcohol-Induced Psychotic Disorder, With Hallucinations  291.4 PATHOLOGIC ALCOHOL INTOX  291.5 Alcohol-Induced Psychotic Disorder, With Delusions  291.8 ALCOHOLIC PSYCHOSIS NEC*  291.81 Alcohol Withdrawal  291.82 Alcohol-Induced Sleep Disorder  291.89 Alcohol-Induced Mood Disorder; Alcohol-Induced Sexual Dysfunction; Alcohol-Induced Sleep Disorder; Alcohol-Induced Anxiety Disorder  291.9 Alcohol-Related Disorder NOS  292 DRUG PSYCHOSES*  292.0 Amphetamine, Cocaine, Nicotine, Opioid, or Other (or Unknown)] Withdrawal; Sedative, Hypnotic or Anxiolytic Withdrawal  292.1 DRUG PARANOID/HALLUCINOS*  292.11 Amphetamine, Cannabis, Cocaine, Hallucinogen, Inhalant, Opioid, Phencyclidine, or Other (or Unknown) Substance-Induced Psychotic Disorder, With Delusions; Sedative, Hypnotic or Anxiolytic-Induced Psychotic Disorder, With Delusions  292.12 Amphetamine, Cannabis, Cocaine, Hallucinogen, Inhalant, Opioid, Phencyclidine, or Other (or Unknown)] Substance-Induced Psychotic Disorder, With Hallucinations; Sedative-, Hypnotic- or Anxiolytic-Induced Psychotic Disorder, With Hallucinations  292.2 PATHOLOGIC DRUG INTOX  292.8 OTHER DRUG MENTAL DIS*  292.81 Amphetamine, Cannabis, Cocaine, Hallucinogen, Inhalant, Opioid, Phencyclidine, or Other (or Unknown)] Substance Intoxication Delirium; Sedative, Hypnotic or Anxiolytic Intoxication or Withdrawal Delirium  292.82 Inhalant or Other (or Unknown) Substance-Induced Persisting Dementia; Sedative-, Hypnotic- or Anxiolytic-Induced Persisting Dementia  292.83 Other (or Unknown)] Substance-Induced Persisting Amnestic Disorder; Sedative-, Hypnotic- or Anxiolytic-Induced Persisting Amnestic Disorder  292.84 Amphetamine, Cocaine, Hallucinogen, Inhalant, Opioid, Phencyclidine, or Other (or Unknown) Substance-Induced Mood Disorder; Sedative-, Hypnotic- or Anxiolytic-Induced Mood Disorder  292.85 Amphetamine, Caffeine, Cocaine, Opioid, or Other (or Unknown) Substance-Induced Sleep Disorder, Sedative-, Hypnotic- or Anxiolytic-Induced Sleep Disorder  292.89 Substance-Induced Anxiety Disorder, Sexual Disfunction, Sleep Disorder, or Intoxication (Refer to the DSM-IV-TR); Hallucinogen Persisting Perception Disorder  292.9 Amphetamine, Caffeine, Cannabis, Cocaine, Hallucinogen, Inhalant, Nicotine, Opioid, Phencyclidine, or Other (or Unknown) Substance-Related Disorder NOS; Sedative-, Hypnotic- or Anxiolytic-Related Disorder NOS |
