# Supplementary material for: Evaluating the effectiveness of concurrent opioid agonist treatment and physician-based mental health services for patients with mental disorders in Ontario, Canada
Source: PLoS One. 2020 Dec 18;15(12):e0243317. doi: 10.1371/journal.pone.0243317 (PMC7748134; doi:10.1371/journal.pone.0243317)
Supplement: S3 Table — (DOCX) [file pone.0243317.s003.docx]

S3 Table: Study outcomes related to Concurrent Physician-Base Mental Health Services and OAT

| **Treatment Groups** |  |  |  |
| --- | --- | --- | --- |
|  | *Patients Weighted N* | *Outcomes Weighted N (%)* | *OR (95%CI)* |
| **All-Cause Mortality** |  |  |  |
| Control (no OAT)* | 753.5 | 90.5 (11.9) |  |
| Tx1 (OAT only) | 18,609.70 | 848.9 (4.6) | 0.4 (0.3-0.4) |
| Tx2 (OAT+psych) | 3,912.20 | 218.1 (5.6) | 0.4 (0.3-0.6) |
| Tx3 (OAT+ MH PC) | 19,266.10 | 937.4 (4.9) | 0.4 (0.3-0.5) |
| Tx4 (OAT+psych+MH PC) | 5,867.80 | 651.2 (6.0) | 0.5 (0.5-0.6) |

| **Treatment Groups** |  |  |  |  |
| --- | --- | --- | --- | --- |
| **ED visits** | *Patients Weighted N* | *Mean (Std Dev)* | *Point Estimate (negative Binomial Regression)* | *OR (95%CI)* |
| Control (no OAT)* | 753.5 | 37.2 (61.2) |  |  |
| Tx1 (OAT only) | 18,609.70 | 19.4 (25.6) | -0.7 | 0.1 (0.0-0.2) |
| Tx2 (OAT+psych) | 3,912.20 | 33.4 (43.4) | -0.1 | 0.9 (0.8-0.9) |
| Tx3 (OAT+ MH PC) | 19,266.10 | 28.5 (35.4) | -0.3 | 0.7 (0.7-0.8) |
| Tx4 (OAT+psych+MH PC) | 5,867.80 | 48.2 (83.1) | -0.2 | 1.3 (1.2-1.4) |
| **Hospitalizations** |  |  |  |  |
| Control (no OAT)* | 753.5 | 12.04 (15.2) |  |  |
| Tx1 (OAT only) | 18,609.70 | 4.97 (6.1) | -0.89 | 0.6 (0.0-0.7) |
| Tx2 (OAT+psych) | 3,912.20 | 6.15 (8.8) | -0.67 | 0.4 (0.2-0.9) |
| Tx3 (OAT+ MH PC) | 19,266.10 | 5.48 (6.3) | -0.79 | 0.8 (0.7-0.9) |
| Tx4 (OAT+psych+MH PC) | 5,867.80 | 8.24 (20.2) | -0.38 | 0.5 (0.4-0.6) |

*N = number of patients*

*Std Dev = Standard Deviation*

*OR = Odds Ratio*

*CI = Confidence Interval*

*Tx = treatment group*

*OAT = opioid agonist treatment*

*Psych = mental health services from a psychiatrist*

*MH PC = mental health services from a primary care physicna*
