# Supplementary material for: Evaluating the effectiveness of concurrent opioid agonist treatment and physician-based mental health services for patients with mental disorders in Ontario, Canada
Source: PLoS One. 2020 Dec 18;15(12):e0243317. doi: 10.1371/journal.pone.0243317 (PMC7748134; doi:10.1371/journal.pone.0243317)
Supplement: Attachment — Submitted filename: Response to reviewers.docx [file pone.0243317.s004.docx]

| **Reviewer question/comment** | **Author Response** | **Reference in manuscript** |
| --- | --- | --- |
| Thank you for stating the following in the Competing Interests section:  [Dr. David Marsh maintains the following roles: Chief Medical Director at CATC (Canadian Addiction Treatment Center), opioid agonist therapy provider. Dr. Marsh has no ownership stake in the CATC as a stipendiary employee. We do not foresee any conflict of interest as data will be made freely available to the public and neither the CATC, nor the Universities, have the ability to prevent publication and dissemination of knowledge. The other authors have no conflicts declared. ].  Please confirm that this does not alter your adherence to all PLOS ONE policies on sharing data and materials, by including the following statement: "This does not alter our adherence to  PLOS ONE policies on sharing data and materials.” (as detailed online in our guide for authors http://journals.plos.org/plosone/s/competing-interests).  If there are restrictions on sharing of data and/or materials, please state these. Please note that we cannot proceed with consideration of your article until this information has been declared.  Please include your updated Competing Interests statement in your cover letter; we will change the online submission form on your behalf. | I have outlined the changes to the acknowledgements and competing interest statement in the cover letter |  |
| Please ensure that you refer to Figure 4 in your text as, if accepted, production will need this reference to link the reader to the figure. | We added a reference to figure 4 |  |
| Please upload a copy of Figure 5, to which you refer in your text on page 20. If the figure is no longer to be included as part of the submission please remove all reference to it within the text. | This was a typographical error and has been removed. |  |
| Please include captions for your Supporting Information files at the end of your manuscript, and update any in-text citations to match accordingly. Please see our Supporting Information guidelines for more information: http://journals.plos.org/plosone/s/supporting-information. | I added a list of supporting files at the end of the manuscript and in-text citations to match |  |
| The [PLOS Data policy](http://www.plosone.org/static/policies.action#sharing) requires authors to make all data underlying the findings described in their manuscript fully available without restriction, with rare exception (please refer to the Data Availability Statement in the manuscript PDF file). The data should be provided as part of the manuscript or its supporting information, or deposited to a public repository. For example, in addition to summary statistics, the data points behind means, medians and variance measures should be available. If there are restrictions on publicly sharing data—e.g. participant privacy or use of data from a third party—those must be specified. | All the data we used was from a third party administrative datasets. We have shared all of the data possible under privacy regulations for Data Analytic Services at ICES. |  |
| **Reviewer 1** |  | ****Note: “in-text reference” is given to allow reviewers to easily find changes made with the search function in the text** |
| Novelty. The conclusions of the manuscript largely agree with consensus literature, which the authors highlight throughout the text. Ultimately, the conclusion appears to be that concurrent disorder treatment improves outcomes for people with OUD? If I am incorrect on this, my apologies. But to that end, I am not sure what it is adding because research must add something to the pool to justify publication, etc. | We want to thank the reviewer for the comment relating to the novelty of the study. We think this prompted us to clarify the rationale for the study.  In the review of the literature, we found that there is contention as to whether concurrent treatment improves outcomes or not. Please see two recent systematic reviews on the topic (Amato, Minozzi, Davoli, & Vecchi, 2011; Dugosh et al., 2016).  We believe that the results of these systematic reviews are due to several methodological limitations. For instance,   - Most of the studies in the existing literature only evaluate methadone as a form of OAT; however, the use of buprenorphine/naloxone as a form of OAT is rising, especially in northern Ontario. - Many authors have previously focused on psychosocial services, but there are limited studies on physician-based mental health services. - Many other studies focus on individual clinical settings rather than population-wide data. This is important because OAT varies significantly across different settings (i.e.), so systematic reviews compare inconsistent control groups. - Lastly, the outcomes evaluated in many studies focus on individual clinical outcomes, such rather than population health outcomes.   This study's novelty lies within connecting the pieces and filling in the gaps that currently exist in the literature. More specifically, the inclusion of suboxone in the primary cohort, the large sample of patients which represents all OAT patients in Ontario, the inclusion of physician-base mental health services that are typically excluded from studies, and the evaluation of broad health outcomes such as health system use (emergency department visits and hospitalizations). | Introduction, line 85-100, in-text reference: *“There is debate in the literature about whether or not adding…”* |
| Heterogeneity. One of the strengths of the study is the large sample size. With the advent of large, administrative datasets, like those of IC/ES, it is possible to conduct gigantic observational studies, and one can use propensity score methods to adjust, quite well. However, an issue remains related to heterogeneity. Despite how close the authors are able to approximate the two populations, I question whether this is a realistic approach given the substantial heterogeneity in the OUD population. As well, I am not sure how useful it is to psychiatrists, clinicians, or addiction medicine specialists if there is limited emphasis on "what" concurrent disorder treatment is helpful, and for "which" concurrent disorders. | We want to thank the reviewer for this comment. We do agree that the OUD population is heterogeneous. However, in Ontario, mental health and OAT are funded differently, and there is little integration or coordination between both types of services. Therefore, the intention of the study was to demonstrate the importance and impact of how patients who do receive concurrent care do better to encourage change at the policy level to incentivize integration of the two types of services. We believe this study can be useful for physicians to demonstrate the importance of concurrent care.  We were also interested in further stratifying patients by type of mental health diagnosis, but this was not possible given the numbers became too small for analytical and reporting purposes after matching. | Introduction, line 80-83, in-text reference: *“In Ontario, mental health and OAT are funded differently and there…”* |
| Secondary data use. This relates to the above point, which stems on the use of secondary data. IC/ES uses a number of proxy measures to estimate exposures and outcomes from administrative datasets. This is akin to handing receipts to an accountant and asking them to determine the financial narrative of someone's life. There are advantages, and disadvantages to this approach, as highlighted by the authors. The main disadvantage the authors encounter is a lack of context, as the study provides a very narrow view of a very complex issue. In order to ensure that data fits a model and to agree with statistical assumptions, many outliers and other aspects of the data have to be trimmed and curtailed. To that end, we see in the flow diagrams that a large sample is significantly cut down by the end. While the authors discuss the limitations, highlighting them is not enough. To that end, there are some serious methodological issues with several of the variables used. For example, using OHIP billing as a proxy for a diagnosis or for providing a treatment for a concurrent disorder is very problematic, as the billing system is raked with discrepancies between what is billed for and what is done. This will never be captured in a study because studies will only cite accuracy of the system; however, this is not a realistic measure of the quality of the data. With that said, I am not sure what a superior method for this would look like that would provide as large of a sample size. Ultimately, many of these issues are generalized limitations of using large administrative data sets to answer clinical questions and unfortunately, the authors have not providing a convincing rationale that there approach is justified given these limitations. | Thank you to the reviewer for this comment. As we indicated, we understand that there are limitations to using secondary data. However, administrative studies allow researchers to quickly conduct cohort studies and examine patient outcomes across healthcare settings. It is important to note that this study is a population health study and not a clinical study. A study involving such a high number of patients across health care settings would otherwise take years if done prospectively. Without administrative data studies, we would not be able to examine such broad health outcomes.  ICES scientists have published hundreds of studies using OHIP billing. Additionally, several studies have evaluated the validity of billing data for research (Davis, Sudlow, & Hotopf, 2016; Lix et al., 2016; Schwartz et al., 2016) and (<https://www.ices.on.ca/Publications/Search-Results?keyword=Physician+billing>).  Additionally, I want to clarify that we used OHIP billing data to identify health services and used diagnosis codes (ICD9 and 10) to determine diagnoses. I have clarified this in the methods. Moreover, we had a patient and family advisory group and two addiction medicine physicians, a psychiatrist, a psychotherapist and a psychologist on the research team. The advisory group helped provide context to the study, and the health professionals consulted helped to understand and contextualize the billing codes used for the research.  The reviewer did not provide any specific changes or questions to address. We hope that the additional rationale and references we added to the manuscript address the reviewer's concerns. | See appendix A for mental health diagnosis code (ICD9 and 10).  Methods, line 126-130, in-text reference: *“A published validation study by Davis et al. found that reporting of mental diagnoses have a strong likelihood…”*  Methods, line 114-121, in-text reference: *“A Patient and Family Advisory Group played a key role in the project…”* |
| Generalizability. My other concern is that the study appears to exclude the highest risk people with severe OUD and severe mental health disorders, which are not "in the system" so to speak. Those who do not have OHIP/Ontario ID, who are marginally housed, who live on reserves, who live in rural and remote regions of Ontario, are left out of the administrative dataset access. Most clinician would argue that the population excluded by this study is the precise population for whom the least is known. Thus, I wish that there was a way to capture those who are understudied, as that would certainly be a novel area for researchers to explore, particularly with the types of methods the authors present. I think this points to a serious issue with selection bias, which limits the generalizability of the authors results; this is particularly worrisome given that the study was already limited in what it could contribute to the literature given that this topic is fairly well-studied. As well, research that presents a more realistic, "real-world" picture of a clinical scenario is more valuable, so, the pertinent exclusions from this study make me wonder if the population that this study explored even "exists" in everyday clinical practice. With this in mind, concurrent substance use disorder is a key comorbidity (e.g., in addition to OUD, stimulant, benzodiazepine, alcohol, etc.), and I am not sure if the authors have appropriately considered this aspect in the analyses. I think this would have a major impact on not only response to OAT, but also, the response to concurrent mental health treatment (as well as the approach to treatment). | The reviewer raises several valuable points here.   1. **Selection bias:** Selection bias is defined as selecting patients that are not representative of the population intended to be analyzed. However, the population we intended to analyze for this study was OAT patients only. We agree that it is of value to study people who do have not accessed OAT care. However, the methods used in this study would not have been appropriate to conduct research involving the populations the reviewer is suggesting. Therefore it was ultimately outside of the scope of this project**.** We do agree that more research is needed on vulnerable populations. 2. **“Real-world” picture:** One of the novel aspects of this project is that it does provide a real-world picture of the OAT population because we included ALL OAT patients in Ontario over the span of 10 years. This is compared to other studies which only represented one or two clinical settings. 3. **Concurrent substance use:** In the OHIP database, substance dependence falls under one category, under one code (304). All patients in the cohort had that code based on their opioid dependence to be included in the study. Therefore, there was no way to detect those who also had a dependence on cocaine, benzodiazepines, or other substances. For this reason, we chose to exclude substance use disorders from our mental disorder diagnosis definition. | Title *“…Concurrent Opioid Agonist Treatment…”*  Abstract, in-text reference: ***“…****for individuals enrolled in Opioid Agonist Treatment in Ontario, Canada. “*  Methods, line 136-141, in-text reference: *“In the OHIP database, substance dependence falls...”* |
| I do not necessarily disagree with the findings, but, I think re-packaging a fairly well-studied topic justifies a higher expectation for methodological rigour and requires researchers to develop innovative strategies to approach the limitations. | We hope that the additional rationale and references we added to the manuscript address the reviewer's concerns. |  |
| **Reviewer 2** |  |  |
| More discussion of the types of commonly co-occurring mental disorders, and the types of complexities that can result (e.g., medical symptom exacerbation, treatment challenges, etc) would strengthen the introduction and clarify the hypotheses (lines 63-66). | We thank the reviewer for this comment. We agree that more information has helped strengthen the introduction and clarify the hypothesis. | Introduction, line 82-88, in0text reference: *“People with opioid use disorder often present with a complex profile of health…”* |
| Rationale for only including mental health care provided by psychiatrists and/or family physicians is needed. Why is physician-based mental health care examined but not other types? | We want to thank the reviewer for allowing us to clarify this. We only included physician-based mental health care because there is very little literature on this topic and it is very common for patients in Ontario, especially in rural areas, to access health care from physicians ("Canadian Mental Health Association. Rural and Northern Community Issues in Mental Health," 2009) | Introduction, line 91-94, in-text reference: *“There is debate in the literature about whether or not adding…”* |
| Rationale for the covariates also needs to be provided. What is the scientific rational for including these medical conditions, but not others? | We want to thank the reviewer again for allowing us to clarify this. The baseline variables were chosen based on previous literature, on the advice of our Patient and Family Advisory Group and on the data available in the databases used for this study. | Methods, line 114-121, in-text reference: *“A Patient and Family Advisory Group played a key role in the project…”*  *Methods, line 204-205, in-text reference: “The baseline variables were chosen…”* |
| Results: the types of co-occurring mental disorders should be described. | Thank you to the reviewer for identifying this shortfall. We have added a section to the results. We also identify and described in Appendix A. | Results, line 267-270, in-text reference: ***“****The most prevalent mental disorders identified in the cohort…”* |
| Line 287: Please cite the work referred to in the statement, “Similar to findings in other studies…” | References have been added. | Discussion, line 323 |

References:

Amato, L., Minozzi, S., Davoli, M., & Vecchi, S. (2011). Psychosocial combined with agonist maintenance treatments versus agonist maintenance treatments alone for treatment of opioid dependence. *Cochrane Database Syst Rev*(10), CD004147. doi:10.1002/14651858.CD004147.pub4

Canadian Mental Health Association. Rural and Northern Community Issues in Mental Health. (2009). Retrieved from <http://ontario.cmha.ca/public_policy/rural-and-northern-community-issues-in-mental-health/#.V9rIno-cFlY>]

Davis, K. A., Sudlow, C. L., & Hotopf, M. (2016). Can mental health diagnoses in administrative data be used for research? A systematic review of the accuracy of routinely collected diagnoses. *BMC Psychiatry, 16*, 263. doi:10.1186/s12888-016-0963-x

Dugosh, K., Abraham, A., Seymour, B., McLoyd, K., Chalk, M., & Festinger, D. (2016). A Systematic Review on the Use of Psychosocial Interventions in Conjunction With Medications for the Treatment of Opioid Addiction. *J Addict Med, 10*(2), 91-101. doi:10.1097/ADM.0000000000000193

Lix, L. M., Kuwornu, J. P., Kroeker, K., Kephart, G., Sikdar, K. C., Smith, M., & Quan, H. (2016). Estimating the completeness of physician billing claims for diabetes case ascertainment using population-based prescription drug data. *Health Promot Chronic Dis Prev Can, 36*(3), 54-60.

Schwartz, K. L., Jembere, N., Campitelli, M. A., Buchan, S. A., Chung, H., & Kwong, J. C. (2016). Using physician billing claims from the Ontario Health Insurance Plan to determine individual influenza vaccination status: an updated validation study. *CMAJ Open, 4*(3), E463-E470. doi:10.9778/cmajo.20160009
