# Supplementary material for: Evaluating the effectiveness of concurrent opioid agonist treatment and physician-based mental health services for patients with mental disorders in Ontario, Canada
Source: PLoS One. 2020 Dec 18;15(12):e0243317. doi: 10.1371/journal.pone.0243317 (PMC7748134; doi:10.1371/journal.pone.0243317)
Supplement: Attachment — Submitted filename: Response to reviewers.docx [file pone.0243317.s005.docx]

Dear reviewers and editorial team,

Thank you for your thoughtful review. We sincerely believe that the revisions have resulted in a stronger manuscript. Please see our response and the reference to the changes in the manuscript summarized in the table below.

| **Reviewer question/comment** | **Author Response** | **Reference in manuscript** |
| --- | --- | --- |
| Reviewer #2: The revisions have resulted in a stronger manuscript. Please see below for remaining issues.  Line 63-66. Although authors do describe psychosocial and health problems in the next paragraph, information on the types of mental disorders referred to remains missing (e.g., depression, anxiety, psychotic disorders, etc) | Thank you for highlighting. We agree that this is a very important issue to highlight | Manuscript track changes, line 63-70, in-test reference: ***“****The most prevalent mental disorders reported in this population are anxiety and mood disorders, including major depression and bipolar disorder. It is essential to highlight the potential underestimation of the prevalence of post-traumatic stress disorder (PTSD) since it is known to be often misdiagnosed as another anxiety, depression or other related disorders.”* |
| Line 146, the use of 1st person is out of place. | I agree and have made the change | Manuscript track changes, line 150, in-text reference: ***“****For this reason, we chose to…”* |

Kindest regards,

Dr. David C. Marsh
